# Flexor Injury Rehabilitation Splint Trial (FIRST): protocol for a pragmatic randomised controlled trial comparing three splints for finger flexor tendon repairs

**DOI:** 10.1186/s13063-024-08013-z

**Published:** 2024-03-16

**Authors:** Emma Bamford, Hannah Berntsson, Suzanne Beale, Lauren Desoysa, Joseph Dias, Sienna Hamer-Kiwacz, Daniel Hind, Nick Johnson, Amanda Loban, Kaye Molloy, Emma Morvan, Ines Rombach, Anna Selby, Praveen Thokala, Chris Turtle, Stephen Walters, Avril Drummond

**Affiliations:** 1 Pulvertaft Hand Centre, Royal Derby Hospital, University Hospitals of Derby and Burton NHS Foundation Trust, Derby, DE22 3NE UK; 2 SCHARR, Division of Population Health, School of Medicine and Population Health, University of Sheffield, S1 4DA, Sheffield, UK; 3 Queen Elizabeth Hospital, University Hospitals Birmingham NHS Foundation Trust, B15 2GW, Birmingham, UK; 4 University Hospitals of Leicester NHS Foundation Trust, LE1, 7RH, Leicester, UK; 5 Derby, England; 6 School of Health Sciences, University of Nottingham, Nottingham, NG7 2QL UK

**Keywords:** Finger flexor tendon rupture, Finger flexor tendon repair, Rehabilitation, Splinting, Hand therapy, Physiotherapy, Occupational therapy, Surgery, Randomised controlled trial

## Abstract

**Background:**

Without surgical repair, flexor tendon injuries do not heal and patients’ ability to bend fingers and grip objects is impaired. However, flexor tendon repair surgery also requires optimal rehabilitation. There are currently three custom-made splints used in the rehabilitation of zone I/II flexor tendon repairs, each with different assumed harm/benefit profiles: the dorsal forearm and hand-based splint (long), the Manchester short splint (short), and the relative motion flexion splint (mini). There is, however, no robust evidence as to which splint, if any, is most clinical or cost effective. The Flexor Injury Rehabilitation Splint Trial (FIRST) was designed to address this evidence gap.

**Methods:**

FIRST is a parallel group, superiority, analyst-blind, multi-centre, individual participant-randomised controlled trial. Participants will be assigned 1:1:1 to receive either the long, short, or mini splint. We aim to recruit 429 participants undergoing rehabilitation following zone I/II flexor tendon repair surgery. Potential participants will initially be identified prior to surgery, in NHS hand clinics across the UK, and consented and randomised at their splint fitting appointment post-surgery. The primary outcome will be the mean post-randomisation score on the patient-reported wrist and hand evaluation measure (PRWHE), assessed at 6, 12, 26, and 52 weeks post randomisation. Secondary outcome measures include blinded grip strength and active range of movement (AROM) assessments, adverse events, adherence to the splinting protocol (measured via temperature sensors inserted into the splints), quality of life assessment, and further patient-reported outcomes. An economic evaluation will assess the cost-effectiveness of each splint, and a qualitative sub-study will evaluate participants’ preferences for, and experiences of wearing, the splints. Furthermore, a mediation analysis will determine the relationship between patient preferences, splint adherence, and splint effectiveness.

**Discussion:**

FIRST will compare the three splints with respect to clinical efficacy, complications, quality of life and cost-effectiveness. FIRST is a pragmatic trial which will recruit from 26 NHS sites to allow findings to be generalisable to current clinical practice in the UK. It will also provide significant insights into patient experiences of splint wear and how adherence to splinting may impact outcomes.

**Trial registration:**

ISRCTN: 10236011

**Supplementary Information:**

The online version contains supplementary material available at 10.1186/s13063-024-08013-z.

## Background

### Background and rationale {6a}

#### The personal and economic burden resulting from finger flexor tendon injuries

Hand injuries in the UK have increased by 57% in 15 years, accounting for 20% of emergency presentations [1]. There were 7346 flexor tendon injuries in 2018–2019; 75% were in working age men [2]. Such injuries most frequently occur from a direct laceration to the tendon in the finger or palm of the hand. Without surgical repair and rehabilitation, divided tendons do not heal; patients cannot bend fingers, grip objects, or effectively care for themselves and others. Long-term prognosis can be poor: 50% of patients report pain and functional limitations 10 years post-injury [3].

#### Flexor tendon repair requires optimal rehabilitation, but the evidence base is limited

The outcome of flexor tendon surgery relies on effective rehabilitation [4]. Patients routinely attend weekly appointments for up to 3 months after surgery, with full recovery (when achieved) taking up to 1 year [5]. There are two components to rehabilitation: exercises to prevent hand stiffness and promote tendon glide/excursion and the provision of a splint to protect the repair.

In the UK, there are three main custom-made splints used in the rehabilitation of zone I/II flexor repairs; however, there is insufficient evidence to inform the efficacy of these splints. A systematic review comparing rehabilitation following flexor tendon surgery suggested that future high-quality randomised controlled trials were required to establish which rehabilitation regimes are safe and most effective [6].

### Long—forearm and hand-based splint

A survey of UK current practice used to inform the feasibility of FIRST found that the long splint is the most commonly used splint following flexor tendon repairs. This has been the mainstay of clinical practice in the UK since the 1980s. This splint protects the newly repaired tendons by preventing movement at the wrist and reducing extension of the fingers.

### Short—hand-based splint

This was developed to allow combined wrist and finger movement and is believed to reduce the risk of complications such as stiffness, fixed flexion deformities of the interphalangeal joints (IPJs), and tendon adhesions due to the increased excursion of the repaired tendons through the synergistic motion of the wrist and hand. This has been reported in a case-series comparing the short and long splint for zone I/II flexor repairs [7]. Our recent survey showed that the short splint has been incorporated into rehabilitation regimes for zone I/II repairs in approximately 50% of hand centres across the UK.

### Relative motion flexion splint (Mini)—finger-based splint

Over the last 5 years, there has been a worldwide shift in the management of extensor tendon repairs moving from using a long splint to the relative motion extension splint (RMES) [8]. More recently, the concept of relative motion splinting has also been introduced into clinical practice for flexor tendon repairs. The relative motion flexion (RMFS (mini)) splint positions the affected digit into relative flexion and therefore utilises the quadriga effect to reduce the biomechanical pull on the repaired tendon. It is also worn in combination with a wrist splint for the first 3 weeks in order to limit full extension of the wrist. The mini splint is thought to provide less protection than the long and short splints described above, but the patients’ function may be improved whilst wearing the splint. This may lead to reduced joint stiffness.

### Rationale for current trial

Patients and clinicians need to know if one splint provides superior outcomes in terms of pain and function: this is the main justification of conducting this RCT. The process evaluation will help us understand how patient-level factors moderate adherence and how adherence mediates benefit and harm outcomes, which is critical for decision-making. Economic evaluation is essential because, while splint costs are comparable and relatively small, the costs of treatment failure and reintervention are substantial. The trial will be conducted in accordance with the protocol and the International Conference on Harmonisation (ICH) Good Clinical Practice (GCP).

### Objectives {7}

#### Aims

The aim is to investigate the clinical and cost-effectiveness of three splints, and mediators of effectiveness, in the repair of zone I/II finger flexor tendons to determine superiority.

#### Objectives

- To determine if any splint is superior in terms of a patient-rated measure of pain and function
- To investigate how patient values and splint acceptability moderates objectively measured splint adherence and how adherence mediates effectiveness
- To evaluate splint cost-effectiveness, from an NHS and societal perspective

### Trial design {8}

This is a parallel group, three-arm, superiority, analyst-blind, multi-centre, individual participant-RCT.

Consenting participants will be randomised to receive either the long splint, short splint, or mini splint. Outcome data will be collected at 6, 12, 26, and 52 weeks post-randomisation.

An 8-month internal pilot will assess the feasibility of the RCT. The progression criteria will be applied to data collected 8 months after the first site is opened. The progression criteria (see Table 1) will be assessed by the trial steering committee (TSC) at the end of the following month.

**Table 1:** Progression criteria

Criterion	Red (% complete)	Amber (% complete)	Green (% complete)
Number of Sites opened	< 15 (75%)	> = 15 (75%) and < 20	20 (100%)
Rate/site/month	< 0.9 (< 60%)	> = 0.9 (60%) and < 1.4	1.5 (100%)
Number of participants recruited	< 144 (< 60%)	> = 144 (60%) and < 240	240 (100%)
Allocation per protocol	< 90%	> = 90% and < 100%	100%
% FU (% of recruited)	< 50%	> = 50% and < 75%	75%

## Methods: participants, interventions, and outcomes

### Trial setting {9}

Participants will be recruited from outpatient hand clinic/therapy services in 26 NHS hospitals across the UK. The hospitals are a mix of city, regional, and teaching hospitals and include a range of sizes (8 large: > 100 flexor tendon injuries treated a year; 7 medium: 50–100 treated a year; 11 small: < 50 treated a year). See Additional file 1 for details of all 26 hospitals.

### Eligibility criteria {10}

#### Inclusion criteria

In order to be eligible for the RCT, the following criteria must all be met at the point of randomisation:

1. Participants aged 16 or over
2. Primary repair of zone I/II finger flexor tendon
3. Surgical repairs according to BSSH guidelines for flexor tendon repairs

#### Exclusion criteria

Patients who meet the following criteria will not be eligible for the RCT:

1. Patients with associated fractures requiring fixation or additional splintage
2. Tendon lacerations involving 3 or more fingers
3. Revascularisation surgery and/or digital nerve reconstructions requiring a nerve graft
4. Presented for treatment more than 3 weeks following the original injury
5. Patients unable to consent or comply with the rehabilitation regime, for example, due to cognitive, psychological or physical disabilities
6. Currently enrolled in another hand trial

### Who will take informed consent? {26a}

#### Participant identification

Patients listed for a planned repair of zone I/II finger flexor tendon, or who have recently undergone surgical repair of zone I/II flexor tendons, will be identified by delegated site staff and provided with a participant information sheet (PIS).

Following surgery, all patients routinely receive a standard care appointment with hand therapy, where decisions regarding their treatment and splint provision are made. Recruitment to the RCT will therefore be aligned with this appointment. Site staff will explain the RCT procedures and answer any questions the patient may have. Prior to consent, eligibility will be confirmed by the research team by completing an eligibility form.

All patients who are approached about the RCT will be recorded on an anonymised screening form with non-identifiable data. Where patients are not interested in the RCT, or are ineligible following surgery, this will be recorded on the screening form.

At RCT set up, an Equality Impact Assessment will be conducted to ensure all patients have equal opportunity to take part. The best practice guidance from The Centre for Black and Minority Ethnic (BME) Health will also be applied.

### Informed consent process

The site research team will confirm patients’ eligibility post-surgery by completing an eligibility form. Eligible patients will be invited to consent to the RCT at their first-hand therapy appointment post-surgery. They will have already been provided with the PIS and will have had time to consider their potential participation. If they are happy to proceed, written consent will be recorded at the clinic visit. Instances where potential participants decline consent will be recorded on an anonymised screening form within the case report form (CRF). Where given, reasons for declining consent will be recorded.

Participant information sheets and consent forms will be translated into approximately seven different languages. Non-English-speaking participants will be given access to an interpreter if required, to answer any questions they may have.

#### Additional consent provisions {26b}

Separate consent will be taken for the qualitative interviews. With patient consent, FIRST qualitative researchers will have access to contact details for the purposes of contact for the qualitative study. Selected participants will be sent an additional information sheet via email. With permission, informed consent will be recorded verbally over video call, at the time of interview by the researchers.

## Interventions

### Explanation for the choice of comparators {6b}

This pragmatic RCT will assess the three main custom-made splints currently used in the rehabilitation of zone I/II flexor repairs: long forearm and hand-based splint, short hand-based splint, and mini finger-based splint.

### Intervention description {11a}

For each type of splint, the splint will be fitted at the first-hand therapy appointment post-surgery.

#### Patients randomised to long splint

The forearm-based early active motion splint, or ‘long splint’, is a custom-made, thermoplastic splint which allows controlled early active movement. It covers the dorsal aspect of the whole hand and forearm, thereby preventing motion of the wrist and allowing for controlled motion of the fingers. The long splint will be prescribed for 5 weeks continuous wear and intermittently for one more week (during the night and in vulnerable situations (e.g. when in public environments or any areas the patient feels at risk of injuring their hand)). Patients are advised not to use their hand for any activities. The splint will be custom-made for the individual participant by the treating hand therapist using a thermoplastic material according to standardised RCT protocol.

#### Patients randomised to short splint

The Manchester short splint, or ‘short splint’, is a custom-made, thermoplastic splint which covers the dorsal aspect of the fingers but allows motion at the wrist. The Short splint will be prescribed for 5 weeks continuous wear and intermittently for one more week (during the night and in vulnerable situations (e.g. when in public environments or any areas the patient feels at risk of injuring their hand)). Patients are advised to only use their unaffected fingers for light activities. The splint will be custom-made for the individual participant by the treating hand therapist using a thermoplastic material according to standardised RCT protocol.

#### Patients randomised to mini splint

The relative motion flexion splint, or ‘mini splint’, is a custom-made finger-based splint which prevents full extension of the injured fingers but allows the hand and fingers to be used for daily activities with wrist support. The finger element will be worn continuously for 5 weeks and intermittently for one more week (during the night and in vulnerable situations (e.g. when in public environments or any areas the patient feels at risk of injuring their hand)). The wrist element will be worn continuously for the first 3 weeks of splint wear and intermittently for three more weeks (during night and in vulnerable situations, e.g. when in public environments or any areas the patient feels at risk of injuring their hand). The mini splint will be custom-made for the individual participant by the treating hand therapist using a thermoplastic material according to standardised RCT protocol. The wrist element may be an off the shelf wrist brace.

#### All splints

Participants will be consented and randomised during their first-hand therapy appointment post-surgery, and splints will be fabricated and fitted at the same visit by the hand therapist. Hand therapists will be trained in the provision of all three splints prior to commencing the RCT. A video of the fabrication and insertion of the sensors for each splint will be available for all treating therapists to refer to, to aid splint provision.

### Criteria for discontinuing or modifying allocated interventions {11b}

Participants may wish to withdraw from RCT treatment, or there may be a clinical need to withdraw the participant, for example, a serious adverse event which prevents the participant from wearing the splint. Participants will be encouraged to continue taking part in the RCT follow-up. Any changes to the splinting protocol will be recorded.

### Strategies to improve adherence to interventions {11c}

Adherence will be measured using a heat sensor (Orthotimer) inserted into each splint. The sensor will be removed from the splint at the 6-week follow-up visit and sent to Sheffield CTRU where the data will be downloaded. Splint adherence will be calculated as the mean actual time divided by target wear time for the first five weeks of prescribed splint usage. Participants will be aware of the monitor but will not have access to the adherence data.

### Relevant concomitant care permitted or prohibited during the trial {11d}

All participants will receive post-surgical rehabilitation which will be tailored to their needs. All treating therapists will be provided with best practice guidance showing the exercise guidelines to be prescribed. This will include active and passive composite flexion exercises and active interphalangeal extension exercises for all splint groups and wrist/finger tenodesis exercises as appropriate for the short and mini splint groups only.

Any other therapy intervention deemed necessary to manage swelling, stiffness, scar adhesions, and pain would be carried out as per local standard care and recorded in participants’ medical notes.

### Provisions for post-trial care {30}

The majority of participant’s will have completed rehabilitation by the end of their participation in the RCT. Any participants who require further treatment at the end of the RCT will be treated as per standard care.

## Outcomes {12}

### Primary outcome/endpoint

The primary outcome is the mean post-randomisation total score of the Patient-Rated Wrist and Hand Evaluation (PRWHE), measured at 6, 12, 26, and 52 weeks post-randomisation. The PRWHE is a 15-item patient-reported outcome for assessing wrist and hand pain/disability on a scale of 0 to 100 (0 = no pain/disability) [9].

### Secondary outcomes/endpoints

Timepoints for secondary outcome data collection will be consistent with primary outcome data collection.

#### Patient-reported secondary outcomes

1. Patient Evaluation Measure (PEM)—patient-reported measure of care received, function, pain and wellbeing [10]
2. Work productivity and activity impairment (WPAI) [11]
3. EuroQoL EQ-5D-5L—health status questionnaire used to derive quality-adjusted life years (QALYs) for the cost-effectiveness analysis [12]
4. Details of any litigation/compensation for injury
5. Global rating of health change (general health compared to previous time point) [13]
6. Preferences for splint attributes (stated and revealed) and splint acceptability (see the “Process evaluation” section)

#### Clinical secondary outcome

1. Active range of movement (AROM) [14]: The AROM of the affected digit(s) will be measured with a finger goniometer according to a standardised protocol. The total active motion (TAM) will be calculated as the total active flexion of the proximal interphalangeal joint (PIPj) and distal interphalangeal joint (DIPj) motion in a composite fist position minus the extension deficit. The Strickland score will then be calculated from this measurement
  $$\textrm{Strickland}=\frac{\left(\textrm{active}\ \textrm{flexion}\ \textrm{PIPj}+\textrm{DIPj}\right)-\left(\textrm{extension}\ \textrm{deficit}\ \textrm{PIPJ}+\textrm{DIPj}\right)\times 100}{175}$$
2. Grip strength: This will be measured using a GripAble handheld dynamometer [15] using a standardised protocol. Three attempts will be made on each hand, and the average of the three will be recorded
3. Splint adherence: assessed using a temperature sensor in the participants’ splint
4. Complications and adverse events

#### Internal pilot outcomes

The progression criteria will be applied to data collected eight months after the first site is opened to determine the feasibility of the RCT continuing. The progression criteria (site set up, participant recruitment, participant allocation per protocol, and follow-up at 6 weeks) will be assessed by the trial steering committee (TSC) at the end of the following month.

Sheffield CTRU will aggregate RCT data to assess the feasibility of the research and intervention protocols based on the feasibility outcomes shown in Table 1.

#### Participant timeline {13}

The participant timeline is shown in Table 2.

**Table 2:** Participant timeline

	Baseline (clinic)	6 weeks (clinic)	12 weeks (clinic)	26 weeks (clinic)	52 weeks (remote)
**Baseline and other covariates**					
Pre-screening form/log (before baseline visit)	X	-	-	-	-
Eligibility form	X	-	-	-	-
Surgery details form	X	-	-	-	-
Informed consent form	X	-	-	-	-
Contact details	X	-	-	-	-
Demographics	X	-	-	-	-
Employment (including sick pay provision)	X	X	X	X	X
Vehicle use	X	-	-	-	-
Randomisation (at baseline)	X	-	-	-	-
**Primary outcome**					
Patient-Rated Wrist and Hand Evaluation (PRWHE)	X	X	X	X	X
**Patient-reported measures**					
Patient Evaluation Measure (PEM)	X	X	X	X	X
Work productivity and activity impairment (WPAI)	X	X	X	X	X
EuroQoL EQ-5D-5L	X	X	X	X	X
Litigation/compensation	-	-	-	-	X
Global rating of change (GRoC)	-	X	X	X	X
Preferences for splint attributes	X	X	-	-	-
**Clinical outcomes**					
Range of movement	X	X	X	X	-
Grip strength	-	-	X	X	-
Splint adherence from heat sensor	-	X	-	-	-
Complications and AE/SAEs	X	X	X	X	X

#### Sample size {14}

The sample size was calculated using the methodology and formula for repeated outcome measures [16]. We assumed the following: (i) 90% power; (ii) 1.67% two-sided significance level (to allow for three head-to-head comparisons between the three randomised groups); (iii) 1 baseline and 4 repeated assessments at 6, 12, 26, and 52 weeks post-randomisation; (iv) a target difference of 6 points [17, 18] in the post-randomisation mean PRWHE scores between any two of three groups; (v) a standard deviation of 20 points for the PRWHE outcome at each post-randomisation time point [9, 16–18]; (vi) an exchangeable correlation or compound symmetry of 0.50 between the repeated PRWHE assessments at 6, 12, 26, and 52 weeks post-randomisation [9, 18]; (vii) 20% attrition. With these input parameters, 114 participants per group are required (3 × 114 = 342 in total). After allowing for 20% attrition, we propose to randomise 429 participants in a 1:1:1 ratio (143 long splint: 143 short splint: 143 mini splint).

#### Recruitment {15}

The anticipated recruitment period is 20 months. The estimated overall recruitment rate is 1.2 per month, with site-specific recruitment rates varying from 0.2 to 3.1. Strategies for achieving adequate recruitment will include training staff on the RCT protocol and providing guidance on how to discuss the RCT with potential participants. A screening log will be completed for non-recruited, potentially eligible patients and monitored closely to identify any recurring reasons for non-consent. We will seek advice from our Patient and Public Involvement and Engagement (PPIE) group, and all patient facing materials used during recruitment will be reviewed by our PPIE group.

### Assignment of interventions: allocation

#### Sequence generation {16a}

Once eligibility has been confirmed, consent acquired, and baseline data taken, the participant will be randomly allocated to either the long splint arm, the short splint arm, or the mini splint arm on a 1:1:1 basis, using a web-based randomisation system provided by Sheffield CTRU. Randomisation allocations will be based on computer-generated pseudo-random lists, stratified by site, with random permuted block sizes.

#### Concealment mechanism {16b}

Randomisation will be completed using a secure, central online randomisation service hosted by the University of Sheffield. Participant ID, site, and confirmation of eligibility and consent will be entered into the randomisation system and the treatment allocation will be confirmed. Allocation concealment will be ensured as the central online randomisation service will not release the randomisation code until the patient has been recruited into the trial.

#### Implementation {16c}

The allocation sequence will be generated by the RCT statistician, who is independent of the participating NHS Trust sites. Randomisation will be done by delegated site staff during the clinic visit and participants will be informed of the outcome verbally. Participants’ GPs will also be informed of their participation in the RCT and their treatment allocation.

## Assignment of interventions: blinding

### Who will be blinded {17a}

In view of the nature of the intervention, patients and their treating clinicians cannot be blinded to treatment allocation. However, to avoid the risk of bias, clinical assessors at sites measuring AROM and grip strength will be blinded to allocation of the participant. The RCT statistician responsible for data analysis will remain blind until the completion of data cleaning. The quality control will be undertaken by an unblinded statistician, who will also attend DMECs, TSCs, and TMGs during the RCT conduct.

### Procedure for unblinding if needed {17b}

Where clinical assessors are inadvertently unblinded, sites will complete an unblinding form and report the unblinding incident to the CTRU RCT manager who will maintain a log of unblinding instances. Site staff will be prompted to record and report any unblinding incidents on the clinical assessment CRFs for each visit.

## Data collection and management

### Plans for assessment and collection of outcomes {18a}

All clinical data will be entered by research site staff onto the CTRU’s in-house data management system (Prospect). Patient-reported outcome measures (PROMs) data (to include the primary outcome assessment) will be completed online by the patient using a tablet in clinic at baseline, 6, 12, and 26 weeks, with paper copies available if this is not possible. PROMs may also be completed remotely via email, post or over the phone where required, if a participant is unable to attend an in person visit. At 52 weeks, all questionnaires will be completed remotely (via email/post or over the phone). 

Complications and adverse events (AEs)/serious adverse events (SAEs) will be assessed at each clinic visit and via phone call at 52 weeks by a delegated member of the research team at each site specific to the participant.

### Plans to promote participant retention and complete follow-up {18b}

Participants who withdraw from RCT treatment will be encouraged to continue as participants in the RCT follow-up. Participants may withdraw their consent to continue with follow-up for the RCT at any time, without providing a reason for this. Any data collected up to the point of the participant’s withdrawal will be retained, and used in the final analysis, and this will be made clear to the patient at the time of consent.

Participants who wish to withdraw from follow-up will be given the option to withdraw only from RCT visits and continue to complete follow-up remotely, where possible. Similarly, participants who do not attend a research follow-up visit(s) may be given the option to complete this follow-up visit remotely. This will ensure lost data is minimised and that the primary outcome is collected in a timely manner. Non-responders to email/postal questionnaires at all time points may be followed up by the CTRU research team using contact details provided by the participant.

The primary outcome is currently collected routinely at some sites, but not at all. Where applicable, if a patient chooses to withdraw from the entire RCT, they will be asked if they are happy for the RCT team to use their routinely collected data in order to inform the primary outcome. This will be optional, but if the patient agrees, it will help to maintain the statistical power and reduce the potential of bias introduced due to missing data when assessing RCT outcomes. Participants with ongoing adverse events at the point of withdrawal will also be asked if they are agreeable for routinely collected data to be used to inform safety outcomes. The RCT team will document this discussion on a RCT specific form and provide the participant with a copy for their records.

Efforts will be made to keep participants engaged in RCT follow-up. Regular updates will be posted on the RCT website and/or communicated via email or newsletter. Vouchers will be provided, and prize draws may take place, for participants who complete follow-up questionnaires. Follow-up visits have been aligned with routine clinic visits where possible, and routine outcome data will be used wherever possible, to minimise the additional burden on participants. Participants will only be considered lost to follow-up if they have not returned the week 52 questionnaires at the point of RCT closure. Where participants do not attend a scheduled appointment, or cannot be contacted to schedule an appointment, questionnaires will be sent via email and contact will be attempted again at each subsequent time point, unless the participant withdraws from the RCT.

### Data management {19} and confidentiality {27}

Participant confidentiality will be respected at all times and the principles of General Data Protection Regulation (GDPR) will be followed. The investigator will ensure that identifiable data is kept securely and protected from unauthorised parties. All patients will be given a unique RCT identifier (participant ID) on entry into the RCT, and this will be used in all future RCT correspondence outside of the direct care team and on data collection forms. Names, email addresses, phone numbers, and addresses where required (if participants prefer to receive paper questionnaires via post) will be collected on the RCT database, to facilitate sending and follow-up questionnaires at the week 52 remote visit, and contacting participants about the qualitative interviews, where consent to do so has been obtained. Access to personal data will be available only to those who need it.

All aspects of data management, including data protection and archiving, will be provided by the University of Sheffield CTRU in accordance with their own standard operating procedures (SOPs). The RCT will use the CTRU’s in-house data management system (Prospect) for the capture and storage of participant data. Project-specific procedures for data management will be detailed in a separate data management plan.

The investigator or delegate at each site will maintain comprehensive and accurate source documents to record all relevant RCT information regarding each participant, in all instances where the database does not form the source data.

### Plans for collection, laboratory evaluation, and storage of biological specimens for genetic or molecular analysis in this trial/future use {33}

Not applicable.

## Statistical methods

### Statistical methods for primary and secondary outcomes {20a} and additional analyses {20b}

Data will be reported and presented according to the CONSORT [19]. All analyses described below will be on the as-randomised (intention to treat) basis, unless specified otherwise. Full details of the statistical analysis will be provided in a separate statistical analysis plan (SAP) which will be finalised prior to the end of follow-up. The SAP will be made available on the trial ISRCTN site. The analysis will be conducted in accordance with CTRU SOPs.

The final analysis will take place after all follow-up has been completed.

Baseline demographic, physical and clinical characteristics, and health-related quality of life data will be described and summarised overall and for the three randomised groups.

### Primary outcome

The primary outcome will be the mean post-randomisation total score on the PRWHE measured at 6, 12, 26, and 52 weeks post-randomisation. The primary effectiveness analysis will compare the post-randomisation PRWHE scores, between the three randomised groups, using a linear mixed model incorporating all post randomisation PRWHE scores (at 6, 12, 26, and 52 weeks) as outcomes, with random effects for centre and subject (to account for the repeated observations per patient), and fixed effects for randomised group, time post-randomisation, and baseline score [20]. We shall assume an exchangeable correlation between the repeated measurements.

Three treatment effect contrasts will be estimated and reported from the linear mixed model: (1) long vs short splint (2); long vs mini splint (3); mini vs short splint. We will estimate 98.3% confidence intervals for the three treatment effects for simultaneous inference and to ensure that all parameters are covered with 95% confidence. This model will include all patients who provide valid PRWHE data for at least one post-randomisation follow-up time point.

### Complications, safety outcomes, and adverse events

The following summaries will be presented: the number and percentages of patients reported as having SAEs in each treatment arm; the number and percentages recorded as having all forms of AEs in each arm; this will be presented as overall and stratified by AE classification. Other complications (e.g. damage to splint, splint modifications) will be presented and reported in a similar way to SAEs.

### Secondary outcomes

The scores on the repeated continuous secondary outcomes (e.g. PEM, EQ-5D-5L, AROM, Grip strength, Splint adherence) will be compared between the randomised groups using a similar longitudinal mixed effects linear regression model as described for the analysis of the primary outcome. Treatment effects for each protocol stipulated follow-up points will also be presented.

Adherence to the randomised splint treatment during the first 6 weeks post-randomisation will be estimated from the heat sensor in the splint. Adherence will be summarised for each randomised group using a variety of summary measures (e.g. mean number of hours per day wearing the splint) and mean adherence compared between the group using a linear regression model. As with the primary outcome, three treatment effect contrasts and their associated confidence intervals will be estimated and reported from the model: (1) long vs short splint, (2) long vs mini splint, (3) mini vs short splint.

### Economic evaluation

The health economic analysis will estimate the costs and QALYs of each of the splints and will be conducted in two parts. First, a within-trial cost-effectiveness analysis (i.e. economic evaluation alongside clinical trial (EEACT)) will be performed, and second, an analysis of the long-term cost-effectiveness will be conducted using a de novo decision analytic model. The cost-effectiveness of three splints will be estimated as incremental cost-effectiveness ratios (ICERs) using full incremental analysis, accounting for any dominance. In the within trial analysis, QALYs will be estimated by calculating the area under the curve for health utility using the EQ-5D-5L, and the costs will be estimated for the health service resource use up to 1 year multiplied by national average costs. Long-term cost-effectiveness modelling will use the data from the RCT (on proportions of patients with complications and adverse events) to estimate the lifetime QALYs and costs. Sensitivity analyses will explore the potential impact of parameters upon costs, QALYs and ICERs. Parameter uncertainty will be included in probabilistic sensitivity analysis based on Monte Carlo simulation. Cost-effectiveness acceptability curves (CEACs) will be plotted to identify the probability of each splint being cost effective for a range of threshold values for an additional QALY.

### Process evaluation

The MRC framework states that process evaluations should inform practice through answering three questions about how interventions work [21]:

1. What is implemented?
2. How does context affect implementation and outcomes?
3. How did the effects of each intervention occur (mechanisms of impact)?

The process evaluation sub-study aims to answer the above questions via the following objectives:

- Collecting data on adherence to splint prescription
- Collecting patient-reported data on (‘stated’) preferences for particular splint attributes, at baseline
- Collecting patient-reported data on (‘revealed’) preferences and splint acceptability at 6 weeks
- Conducting qualitative interviews
- Developing a structural equation model, to show the effect of baselines, preferences, acceptability, and adherence on pain/function (PRWHE: RCT primary outcome)

### Interim analyses {21b}

There is no planned interim analysis, beyond checking the recruitment and retention rate at the end of the pilot phase.

### Methods for additional analyses (e.g. subgroup analyses) {20b}

Subgroup analyses will be performed to explore whether there is heterogeneity in treatment effect for the primary endpoint across the following pre-specified subgroups: sex, employment type, eligibility for occupational sick pay, age category, zone of injury, and tendons repaired.

The subgroup analyses will be performed on the ITT population only. The analysis will be based on the primary analysis model with the addition of an interaction term between the treatment and subgroup to assess the stability of the result in different populations. For simplicity, the interaction between treatment and time will be excluded from this model. Treatment effect estimates for the mean PRWHE post-randomisation scores with 98.3% confidence intervals will be calculated for each sub-group. The results will be displayed graphically using forest plots. No *p*-values will be presented, as it is acknowledged that the trial is not powered for these subgroup analyses, and they are considered exploratory.

### Methods in analysis to handle protocol non-adherence and any statistical methods to handle missing data {20c}

The impact of missing PRWHE outcome data will be minimised to some extent by using the linear mixed model, which allows the inclusion of intermittent responders in the primary analysis. PRWHE scores for complete and intermittent responders will be compared descriptively. The impact of missing data will additionally be assessed using multiple imputation by chained equations (MICE). Missing outcome and covariate data will be predicted by age, rupture rate, hand dominance, available PRWHE data at other follow-up time points, and any baseline covariates found to be predictive of the outcome data. The estimates of the treatment effects and their associated confidence interval from the imputation model will be graphically displayed alongside the results for the observed data. Additional sensitivity analysis will consider scenarios whereby participants with missing data have outcomes worse than those with available data (missing not at random scenarios). The primary analysis approach will be based on the as-randomised population, analysing participants in their randomised groups regardless of adherence to their randomised intervention.

### Plans to give access to the full protocol, participant-level data, and statistical code {31c}

The full protocol is available via this document. Anonymised datasets and statistical code may be available from the corresponding author on reasonable request following completion of the RCT.

## Oversight and monitoring

### Composition of the coordinating centre and trial steering committee {5d}

FIRST will be led by the CI and co-CI working in coordination with the co-applicants and Sheffield CTRU, who will form the Trial Management Group (TMG). The CI will chair TMG meetings to discuss the day to day running of the RCT, including any implementation issues. The TMG will receive reports from the trial steering committee (TSC) and data monitoring and ethics committee (DMEC) to manage trial progress.

The TSC will consist of an independent chair and other professionals with relevant clinical and academic experience and one patient representative. The TSC will meet at regular intervals, as defined in the TSC terms of reference. The TSC can prematurely close the RCT, should this be recommended by the DMEC.

### Composition of the data monitoring committee, its role and reporting structure {21a}

The DMEC will consist of an independent statistician, and at least two independent clinicians with clinical trial expertise. The DMEC will review reports provided by the CTRU to assess the progress of the RCT, the safety data, and the critical endpoint data as required. The DMEC will meet at regular intervals, as defined by the DMEC charter. There will be no interim analyses (other than for the purposes of the blinded internal pilot) or definitive stopping guidelines, but the DMEC will be able to request unblinded data and recommend RCT termination to the TSC/funder on grounds of safety or futility.

### Adverse event reporting and harms {22}

AEs and SAEs will be recorded from the point a participant provides written informed consent for RCT entry and up until participant’s completion of the RCT. Ongoing AE/SAEs will be followed up until the event has resolved or stabilised or until the participant’s involvement in the RCT has ended.

All AEs will be assessed by site staff for relatedness and seriousness (see seriousness criteria in Table 3). Non-serious AEs will only be recorded where they involve the injured hand/upper limb or are considered possibly related to the injury or its treatment. All AEs which meet the criteria for seriousness will be recorded, regardless of relatedness. AEs will be recorded on the adverse event form within the participant CRF and in the medical notes. Sites are asked to enter all available information onto the RCT database as soon as possible after the site becomes aware of the event.

**Table 3:** Adverse event definitions

Term	Definition
Adverse event (AE)	Any untoward medical occurrence in a trial participant
Serious adverse event (SAE)	An AE which is serious, defined as any untoward medical occurrence or effect that: • Results in death • Is life-threatening* • Requires hospitalisation or prolongation of existing inpatients’ hospitalisation** • Results in persistent or significant disability or incapacity • Is a congenital anomaly/birth defect • Is otherwise considered medically significant by the investigator***
Related AE/SAE	An AE or SAE which is related to a research procedure
Unexpected AE/SAE	An AE or SAE which has not been pre-specified as expected
Notable event	An event of particular interest that does not necessarily meet the criteria for seriousness but requires expedited reporting as per the protocol

^*^The term life-threatening in the definition of a serious event refers to an event in which the patient is at risk of death at the time of the event; it does not refer to an event that hypothetically might cause death if it were more severe, for example, a silent myocardial infarction

^**^Hospitalisation is defined as an inpatient admission, regardless of length of stay, even if the hospitalisation is a precautionary measure for continued observation. Hospitalisations for a pre-existing condition, that has not worsened or for an elective procedure do not constitute an SAE

^***^Other important medical events that may not result in death, be life-threatening, or require hospitalisation may be considered a serious adverse event/experience when, based upon appropriate medical judgement, they may jeopardise the patient and may require medical or surgical intervention to prevent one of the outcomes listed in this definition

SAEs will require more detailed information to be recorded. For the purposes of this RCT, flexor tendon rupture is considered a medically significant event, and any incidents will be recorded as SAEs.

SAEs must also be reported to the Sheffield CTRU immediately but within a maximum of 24 h of the site becoming aware of the event, unless exempt. The CTRU will coordinate ongoing monthly reporting to the sponsor or as soon as possible if unexpected SAE. The DMEC and TSC will also receive information on all AEs and SAEs, at a frequency agreed with each committee and documented in the appropriate charter/terms of reference.

The following events are expected and, should they meet the criteria for seriousness, do not require reporting to CTRU within 24 h, but should be reported within the time frames specified below:

Within 72 h, for ongoing safety monitoring purposes:

1. Flexor tendon rupture

Before the participants next scheduled follow-up visit:

2. Local pressure areas because of the splint, plaster of paris, or dressings
3. Infection leading to:
  - Treatment with oral antibiotics
  - Treatment with intravenous antibiotics either as an inpatient or outpatient
  - Requiring surgical washout
4. Stiffness of the affected hand requiring surgery, e.g. tenolysis/arthrolysis
5. Scar issues, e.g. hypersensitivity/hypertrophic scars
6. Delayed wound healing requiring an extended period of dressing
7. Complex regional pain syndrome
8. Fixed flexion deformity of the proximal interphalangeal joint (PIPj) or distal interphalangeal joint (DIPj) requiring additional splintage

SAEs which are deemed related to the research and are not expected will be reported to the research ethics committee (REC) who approved the RCT.

## Frequency and plans for auditing trial conduct {23}

Central and/or on-site monitoring will be undertaken at a level appropriate to the RCT risk assessment and will be documented in the trial monitoring plan. Regular site monitoring visits will occur throughout the RCT as specified in the RCT monitoring plan, and additional visits will be undertaken where required. At these visits, the monitor will review activity to verify the following:

1. Data are authentic, accurate, and complete
2. Safety and rights of the patient are being protected
3. RCT is conducted in accordance with the approved protocol and RCT agreements, GCP, and all applicable regulatory requirements

Accurate and reliable data collection will be assured by verification and cross-check of the CRF against the investigator’s records by the RCT monitor (source document verification). The RCT monitor will contact sites regularly to inspect CRFs throughout the RCT, to verify adherence to the protocol and completeness, consistency, and accuracy of the data being entered on the CRFs.

## Plans for communicating important protocol amendments to relevant parties (e.g. trial participants, ethical committees) {25}

Important protocol amendments will be agreed with the funder, sponsor, and TSC and submitted to the REC and Health Research Authority (HRA) for approval. All amendments will be implemented in accordance with the guidance of the HRA. Where required, RCT participants will be informed in writing of any changes.

## Dissemination plans {31a}

Results of the RCT will be disseminated through peer reviewed scientific journals and at clinical and academic conferences, as well as submission of a final report to the funder, which will be made available online. Details of the RCT will also be made available on the Sheffield CTRU website.

Our PPIE representatives will review our results and support dissemination to patients through video content on professional society webinars and social media. Newsletter and webinar feedback for study participants, about recruitment and results, will be guided by our PPIE. At the close out and write up phase, we will seek PPIE input into the final study report. The results will be published on a freely accessible database within one year of completion of the RCT. To capture and evaluate the PPIE impact, an impact log will be completed by the PPI lead at every PPI activity.

Full details, including guidance on authorship, are documented in the RCT publication and dissemination plan.

## Discussion

FIRST aims to inform rehabilitation practices for flexor tendon ruptures by comparing the effectiveness of the three different splints currently used in standard care, in terms of patient-reported pain and function (assessed by the PRWHE). The results will inform national hand therapy and hand surgery guidelines. Embedded qualitative and health economic analyses will highlight factors influencing splint adherence and any cost-effectiveness differences between the three splints.

FIRST is a pragmatic RCT, which therefore sought to evaluate the study splints in a real-world setting. However, as different study sites had different levels of experience using the FIRST splints, a potential issue for the research was around the ability of therapists to fabricate the splints required at the different sites. To address this, face-to-face splint fitting training sessions are held with hand therapists from participating sites, to ensure consistency of fabrication across the sites. Splint fitting videos were also developed as a resource for site staff to refer back to and for use in training new staff members unable to attend the in-person sessions. Similarly, training videos for measurement of AROM were developed to promote consistency in AROM measurement across sites. Regular PI drop-in sessions are held as an opportunity for the sites to communicate with the RCT management team and each other and ask questions about the RCT protocol.

In addition to providing guidance on the rehabilitation of flexor tendon ruptures, the training described above is an important component of FIRST and has contributed to the impact that FIRST has had both on clinical development and also the opportunities that have been created for hand therapists to be involved in research. Through participation in the RCT, hand therapists from hand centres across the UK have been trained in fabrication of a splint, or splints, that were not previously used in all centres. Additionally, hand therapy teams with differing levels of experience in clinical research have been trained in the delivery of research, supported by the NIHR associate PI scheme.

The hand therapy community’s support for the RCT is clear and there is a high level of engagement from clinical teams. At the time of protocol publication, the 8-month internal pilot data has been reviewed by the RCT oversight committees and funder, who have agreed the RCT is feasible and advised that it should continue to full recruitment as per the current protocol. Additionally, the study oversight committees have raised no concerns about the per group data (including rupture rates) to date.

## Trial status

Protocol version 2.2, 29 June 2023. FIRST opened to recruitment in August 2022 and is anticipated to complete recruitment in 2024 and follow-up in 2025. Trial progress was reviewed against the pilot criteria in May 2023 by the study oversight committees and the funder. FIRST was deemed to be feasible and recommended to continue to completion.

### Supplementary Information

**Additional file 1.** FIRST Study Sites

**Additional file 2.**

## Abbreviations

AE: Adverse event
AROM: Active range of movement
BME: Black and minority ethnic
BSSH: British Society for Surgery of the Hand
CI: Chief investigator
CEAC: Cost-effectiveness acceptability curves
CRF: Case report form
CTRU: Clinical Trials Research Unit
DIPj: Distal interphalangeal joint
DMEC: Data monitoring and ethics committee
EAM: Early active mobilisation
EEACT: Economic evaluation alongside clinical trial
EQ-5D: EuroQol Five Dimensions Questionnaire
FDP: Flexor digitorum profundus
GCP: Good Clinical Practice
GDPR: General Data Protection Regulation
HTA: Health Technology Assessment
HRA: Health research authority
ICH: International Conference on Harmonisation
IPJs: Interphalangeal joints
ICERs: Incremental cost-effectiveness ratios
ISRCTN: International Standard Randomised Controlled Trial Number
MICE: Multiple imputation by chained equations
NIHR: National Institute for Health Research
PEM: Patient Evaluation Measure
PI: Principal investigator
PIPj: Proximal interphalangeal joint
PIS: Participant information sheet
PPIE: Patient and Public Involvement and Engagement
PROMs: Patient-reported outcome measures
PRWE: Patient-Rated Wrist Evaluation
PRWHE: Patient-Rated Wrist and Hand Evaluation
QALY: Quality-adjusted life years
RCT: Randomised controlled trial
REC: Research ethics committee
SAE: Serious adverse event
SAP: Statistical analysis plan
TMG: Trial Management Group
TSC: Trial steering committee
WPAI: Work productivity and impairment
