# Supplementary material for: Flexor Injury Rehabilitation Splint Trial (FIRST): protocol for a pragmatic randomised controlled trial comparing three splints for finger flexor tendon repairs
Source: Trials. 2024 Mar 16;25:193. doi: 10.1186/s13063-024-08013-z (PMC10943783; doi:10.1186/s13063-024-08013-z)
Supplement: Supplementary file 1 — Additional file 1. FIRST Study Sites [file 13063_2024_8013_MOESM1_ESM.docx]

**FIRST Study Sites:**

Barts Health NHS Trust

Royal United Hospitals Bath NHS Foundation Trust

University Hospitals Birmingham NHS Foundation Trust

North Bristol NHS Trust

Cambridge University Hospitals NHS Foundation Trust

Chelsea and Westminster Hospital NHS Foundation Trust

Royal Cornwall Hospitals NHS Trust

University Hospitals Coventry and Warwickshire NHS Trust

University Hospitals of Derby and Burton NHS Foundation Trust

NHS Lanarkshire

Guy’s and St Thomas’ NHS Foundation Trust

Hull University Teaching Hospitals NHS Trust

University Hospitals of Leicester NHS Trust

Newcastle upon Tyne Hospitals NHS Foundation Trust

Norfolk and Norwich University Hospitals NHS Foundation Trust

Northampton General Hospital NHS Trust

Oxford University Hospitals NHS Foundation Trust

Portsmouth Hospitals University National Health Service Trust

Lancashire Teaching Hospitals NHS Foundation Trust

Queen Victoria Hospital NHS Foundation Trust

Royal Free London NHS Foundation Trust

Salisbury NHS Foundation Trust

The Shrewsbury and Telford Hospital NHS Trust

South Tees Hospital NHS Foundation Trust

Buckinghamshire Healthcare NHS Trust

Swansea Bay University Local Health Board
