# Supplementary material for: Flexor Injury Rehabilitation Splint Trial (FIRST): protocol for a pragmatic randomised controlled trial comparing three splints for finger flexor tendon repairs
Source: Trials. 2024 Mar 16;25:193. doi: 10.1186/s13063-024-08013-z (PMC10943783; doi:10.1186/s13063-024-08013-z)
Supplement: Supplementary file 2 — Additional file 2. [file 13063_2024_8013_MOESM2_ESM.pdf]

The University Of Sheffield. Clinical Trials Research Unit.

NHS University Hospitals of Derby and Burton NHS Foundation Trust

FIRST

Prospective randomised controlled trial comparing three splints for finger flexor tendon repairs (FIRST)

Participant Information Sheet

You are being invited to take part in the FIRST study. Before you decide if you would like to take part, it is important for you to understand what taking part involves and why the study is being done. Please take your time to read the following information carefully, discuss it with others and your therapist if you wish. Please ask us if there is anything that is not clear or if you would like more information.

1. What is the research about and what is being tested?

Following flexor tendon repair surgery, all patients are provided with a custom-made splint, which is moulded by a hand therapist in a clinic. The purpose of the splint is to protect the newly repaired flexor tendon. Currently, there are 3 different splints used in the NHS and the use of these splints varies in hospitals across the UK. It is not known which splint is best for patients.

The FIRST study is comparing the three splints that are used in the NHS for patients who are having rehabilitation following flexor tendon repair surgery. We would like to know whether any of the three splints is better at getting patients back to their normal hand use. We would also like to find out what it is like to wear each splint, if patients wear their splints, and whether one splint is better value for money.

2. Why have I been asked to take part?

You may take part because you have either undergone, or are due to undergo, surgical repair of your flexor tendon and will be due to receive a splint as part of your standard care.

3. Do I have to take part?

No. Taking part in this research is always a choice. If you decide not to take part in the study, your care will not be affected. If you do decide to take part, you will be asked to complete a consent form. You are free to change your mind and withdraw from the study at any time should you wish to do so.

4. What will happen if I take part?

At your first hand therapy appointment you will complete questionnaires about your general health, hand function and splint preferences. Your range of movement (flexibility) of your hand will also be assessed.

FUNDED BY NIHR | National Institute for Health Research

Which treatment will I have?

You will be randomly allocated to receive one of the three splints. The randomisation process is done by a computer, and you or your medical team will not be able to influence or change the splint type you receive. Once this has been done, you will be informed of your splint type and your splint will be made and fitted at the same appointment. Your splint will be fitted with a small temperature sensor(s) to detect when the splint is on and off. These sensors are commonly used and are safe. The sensor stores the data about splint wearing which will then be downloaded by the University of Sheffield research team.

What happens after my first hand therapy visit?

You will receive standard NHS care during your recovery, which often involves weekly therapy appointments. Most of the additional study information will be collected at these appointments. You will be asked to complete the research questionnaires at 6 weeks, 3, 6 and 12 months after surgery. You may not need to attend an appointment at 12 months after surgery, so you will be emailed/posted a link to complete the research questionnaires from home instead. If you are unable to attend your other clinic appointments for any reason, you may complete your questionnaires via email/post or with a member of staff over the phone. Your contact details will be shared with researchers from the University of Sheffield so they can coordinate this.

As part of this study you will also have an extra visit to your clinic at 6 months. During this visit your hand movement will be assessed along with your grip strength. This appointment should take about 30 minutes. You will be reimbursed for your travel expenses for this appointment.

The therapist who assesses your hand flexibility and grip strength will not know which splint you have been wearing. This is to ensure their assessment is not influenced in any way.

After the FIRST study has been running for a few months, we would also like to talk to participants about their experiences of wearing their splint. This is so we can better understand the benefits, and any drawbacks, associated with each splint type. If you are happy to be contacted, we may send you more information about this.

5. What are the potential risks and disadvantages in taking part?

If you were not taking part in the study, you would be treated with one of the three splint types. We do not know which of the three splints is best. As such, there are no real harms over and above routine care, linked with taking part in the study.

Taking part in this study will mean an additional appointment at the hospital, compared to the number of appointments you receive normally. Although this means extra travel, you will be reimbursed for your travel costs for attending this appointment if required.

6. What are the potential benefits to taking part?

FUNDED BY NIHR | National Institute for Health Research

If you take part in this study, you will be contributing to important research that will inform treatment choices for patients in future. You will be under close follow-up which is normal for those taking part in research.

7. How will we use information about you?

If you agree to take part, we will need to use information from you and from your medical records for this research project. This information will include your name, NHS number, initials and contact details. People will use this information to do the research or to check your records to make sure that the research is being done properly. People who do not need to know who you are will not be able to see your name or contact details. Your data will have a code number instead. We will keep all information about you safe and secure.

Information collected about you will be stored on a University of Sheffield database. Information collected as part of the study will only be accessible by those involved in the running of the study, including your local hospital, University Hospitals of Derby & Burton NHS Foundation Trust as Sponsor for the study, and researchers at the University of Sheffield who are managing the study and analysing the data. All of these staff are trained in data protection (GDPR).

If you decide to take part, we will inform your GP.

The data from your temperature sensor about how often you wear your splint will be held in a cloud-based system, on a secure encrypted server located in Germany. Data will be held according to the European Data Protection Board (EDPS) guidelines. You will not be identifiable from this data.

We will ask for your postcode, for the purposes of assessing the inclusivity and diversity of our participants population, and for the purposes of exploring whether the area in which you live is related to how well your rehabilitation goes.

Once we have finished the study, we will keep the data for 7 years after the study has finished, so we can check the results. The outcomes of the study may be published in journals, on websites or at conferences, however, you will not be identifiable from the published results, and your personal details will be kept strictly confidential. You will also be asked whether you are happy for anonymised data collected about you to be used in future research, but you will not be told what this research is.

If you agree to take part in the interviews that run alongside this study, we may ask your permission to use anonymised quotes in the published study materials.

University Hospitals of Derby & Burton NHS Foundation Trust is the Sponsor for this study based in the United Kingdom. They will act as the data controller for this study. This means that they are responsible for looking after your information and using it properly.

8. Where can I find out more about how my information is used?

You can find out more about how we use your information

FUNDED BY NIHR | National Institute for Health Research

- At www.hra.nhs.uk/information-about-patients/
- our leaflet available from www.hra.nhs.uk/patientdataandresearch
- by asking one of the research team
- by sending an email to uhdb.dataprotectionofficer@nhs.net, or
- by ringing us on 01332 788 645

9. What happens if I change my mind?

If you decide that you no longer wish to take part, please let your therapist or the research team know. You do not have to give a reason, and this will not impact on the medical care you receive during or after the trial.

Any data collected from you up to the point of withdrawal will be retained and used in the trial results. You will also be asked whether you consent to your routinely collected information to be used for the study, where it is relevant. This means that we will not ask anything of you after your withdrawal, but we may be able to use some of the questionnaires or assessments taken as part of your usual care to help with the trial results.

10. What if there is a problem?

If you have a concern about any aspect of the study, you should ask to speak to the study team, who will do their best to answer any questions you may have. You can contact your local study team via [insert contact details]. Alternatively, you can contact the central study management team at [insert contact]. If you remain unhappy and wish to make a complaint, you can do this through the NHS Complaints Procedure, and the details for your local patient advice and liaison service (PALS) team are available on the study website.

In the event that something does go wrong, and this is due to someone's negligence then you may have grounds for legal action for compensation against your treating hospital, but you may have to pay your legal costs. The normal National Health Service complaints mechanisms will still be available to you (if appropriate).

If you wish to make a report of a concern or incident relating to potential exploitation, abuse or harm resulting from your involvement in this project, please contact the project's Designated Safeguarding Contact, through your local PALS team [insert contact details]. If the concern or incident relates to the Designated Safeguarding Contact, or if you feel a report you have made to this Contact has not been handled in a satisfactory way, please contact the Sponsor safeguarding team via email at uhdb.dataprotectionofficer@nhs.net or phone at 01332 788 645 and/or the University of Sheffield's Research Ethics & Integrity Manager (Lindsay Unwin; l.v.unwin@sheffield.ac.uk).

11. Who is organising and funding the study?

The study has been designed by qualified hand therapists and surgeons, along with patient representatives and researchers. The research is organised by the University of Sheffield Clinical Trials Research Unit on behalf of University Hospitals of Derby & Burton NHS Foundation Trust (the Sponsor).

This project is funded by the NIHR Health Technology Assessment (HTA) Programme (project number NIHR133582).

All research in the NHS is looked at by an independent group of people, called a Research Ethics Committee, to protect your safety, rights, wellbeing and dignity. This study has been reviewed and approved by a Research Ethics Committee.

FUNDED BY
NIHR | National Institute for Health Research

The University Of Sheffield. Clinical Trials Research Unit.

NHS
University Hospitals of Derby and Burton
NHS Foundation Trust

FIRST

Prospective randomised controlled trial comparing three splints for finger flexor tendon repairs **(FIRST)**

Consent Form

Participant Identification Number: S ☐☐ / ☐☐☐

Initial each box

By initialling each box and signing this form:

1. I confirm that I have read the information sheet dated................... (version............) for the above study. I have had the opportunity to consider the information, ask questions and have had these answered satisfactorily. ☐

2. I understand that my participation is voluntary and that I am free to withdraw at any time without giving any reason, without my medical care or legal rights being affected. ☐

3. I understand that relevant sections of my medical notes and data collected during the study may be looked at by individuals from the Sheffield Clinical Trials Research Unit, from regulatory authorities or from the NHS Trust, where it is relevant to my taking part in this research. I give permission for these individuals to have access to my records. ☐

4. I understand that my splint will be fitted with a temperature sensor, to monitor adherence to the splinting protocol. ☐

5. I agree to my GP being informed of my participation in the study and to them being contacted, with my agreement, if the study team have any concerns about my health. ☐

6. I understand that my contact details will be shared with researchers from the Sheffield Clinical Trials Research Unit, for the purposes of contacting me to collect questionnaire responses for the study. ☐

7. I agree that information collected by the research team, including a copy of this signed consent form, can be sent to and stored at the Sheffield Clinical Trials Research Unit for the purposes of monitoring and auditing. ☐

8. I agree that data collected about me without personal identifiers may be used to support other research in the future, and may be shared with other researchers for comparison studies; and I give my permission for this. ☐

9. I agree to take part in the above study. ☐

Please turn over

Optional		**Please tick**
10.	I agree to be contacted by researchers at the University of Sheffield to invite me to take part in an interview about my experience of splinting.	**Yes** ☐ **No** ☐
11.	I agree that researchers can contact me regarding participation in other research. I understand I will be provided with further information and given the opportunity to decide whether or not to participate.	**Yes** ☐ **No** ☐
12.	I would like to receive information about study progress and results.	**Yes** ☐ **No** ☐

Name of Participant | Signature | DD/MM/YYYY

Name of person taking consent | Signature | DD/MM/YYYY

If required:

I confirm that the participant has had access to interpretation in order to give consent.
I have interpreted the above information to the participant to the best of my ability in a way which I believe the participant has understood.

Name of interpreter | Signature | DD/MM/YYYY

1 copy for participant; 1 (original) for site file; 1 copy to be kept with participant's medical notes.

The FIRST Interview Sub-study

A study about patient experiences of wearing splints following flexor tendon repair

Interview Participant Information Sheet

We would like to invite you to take part in an interview about your experience of wearing a splint following flexor tendon repair surgery. Before you decide whether you would like to take part, we want to tell you a bit more about why the research is being done and what it would involve for you. We have provided information below, including how the study will be conducted, to help you decide whether or not you wish to take part. Please take your time to read the information and ask us if anything is unclear.

1. What is this study about?

Following flexor tendon repair surgery, all patients are provided with a custom-made splint, which is moulded by a hand therapist. The purpose of the splint is to protect the newly repaired flexor tendon. Currently, there are 3 different splints used in the NHS and the use of these splints varies in hospitals across the UK. It is not known which splint is best for patients. We are comparing the effectiveness of these three splints in a clinical trial called the FIRST study.

In addition to the clinical trial, we would also like to find out what is important to people in a splint, explore people's experiences of wearing a splint, and see how these impact on how likely someone is to wear their splint. We are interested in this, as adherence to a splint may impact on how effective a splint is.

2. Why have I been invited?

You have been invited to take part in an interview because you have experience of wearing one of the three splints which we are testing in a clinical trial. It is up to you whether you take part, and you can withdraw at any time.

3. What will be involved if I take part?

The research will take the form of an interview, during which we will ask you some questions about yourself and your experience of the splint. Before the interview, the researcher will need to confirm that you are willing to take part. The consent process and interview will last approximately an hour and will be audio recorded. The interview will take place online via video call.

FUNDED BY NIHR | National Institute for Health Research

4. Do I have to take part?

No. It is up to you to decide whether you agree to take part and you can change your mind at any time, without giving a reason. A copy of the consent form will be provided to you. This information sheet is for you to keep.

5. Will the interview be recorded and how will these recordings be used?

With your permission, the interview will be audio recorded using encrypted electronic equipment. The recording will only be available to members of the study team and it will only be used to allow for the preparation of transcripts. Audio recordings will be transcribed by support staff within the University of Sheffield.

6. What will happen to the data and results from the interview?

The anonymised transcript of your interview will be analysed by the FIRST study qualitative researchers. At the end of the research study, we will destroy the audio recordings. The interview transcripts and consent forms will be stored securely for 7 years at the University of Sheffield. If considered appropriate by the research team, the findings from the interviews may be shared in an anonymised form. This could be in written form in research publications or in presentations at conferences.

7. How will we use information about you?

We will need to use information from you for this research project. This information will include your name and contact details. People will use this information to do the research or to check your records to make sure that the research is being done properly. People who do not need to know who you are will not be able to see your name or contact details. Your data will have a code number instead.

Any feedback that we collect during the interview will be treated as confidential and stored securely. The interview transcript may be read by other researchers within the process evaluation team for analysis, but your name will not be included on any transcript. Quotes from the interview may be used in the final report; however, your name will not be included.

We will keep all information about you safe and secure. Once we have finished the study, we will keep some of the data so we can check the results. We will write our reports in a way that no-one can work out that you took part in the study.

What are your choices about how your information is used?

- You can stop being part of the study at any time, without giving a reason, but we will keep information about you that we already have.
- We need to manage your records in specific ways for the research to be reliable. This means that we won't be able to let you see or change the data we hold about you.

You can find out more about how we use your information:

FUNDED BY NIHR | National Institute for Health Research

- At www.hra.nhs.uk/information-about-patients/
- our leaflet available from www.hra.nhs.uk/patientdataandresearch
- by asking one of the research team
- by sending an email to uhdb.dataprotectionofficer@nhs.net, or
- by ringing us on 01332 788 645

8. What are the potential benefits and disadvantages of taking part?

We hope that you will find an opportunity to reflect on your experiences of wearing your splint beneficial and interesting. Your interview data, and those of other participants, will be used to inform clinical practice, with the aim of improving overall patient experience and treatment. We anticipate no major disadvantages to participation other than spending some of your time to complete the interview. We understand that you may need to take a break from or leave the interview at any time. We will take the interview at a time that suits you. We hope that thinking about your experiences will not be distressing but, if that happens, we suggest seeking help from any one or more of the following sources of support and advice:

- Your GP;
- A member of the team involved in your care regarding the splint
- NHS 111;
- The Samaritans (**tel:** 116 123; **email:** jo@samaritans.org)

9. Who is organising and funding this study?

This research is funded by a NIHR HTA grant (Funding Reference NIHR133582). The project is led by University Hospitals of Derby & Burton NHS Foundation Trust and the research study is organised by the University of Sheffield.

All research in the NHS is looked at by an independent group of people, called a Research Ethics Committee, to protect your safety, rights, wellbeing and dignity. This study has been reviewed and approved by a Research Ethics Committee.

10. What if there is a problem?

If you have a concern about any aspect of the study, you should ask to speak to the study team, who will do their best to answer any questions you may have. You can contact your local study team via [insert contact details]. Alternatively, you can contact the central study management team at [insert contact details].

If you would like to make a complaint about the study or have any issues that cannot be resolved with the research team directly, please contact the study's sponsor:

Research & Development Department, University Hospitals Derby & Burton NHS Foundation Trust, Royal Derby Hospital, Uttoxeter Road, Derby DE22 3DT
Email: Uhdb.sponsor@nhs.net

OR

The University’s Research Ethics and Integrity Manager (Lindsay Unwin; l.v.unwin@sheffield.ac.uk).

FUNDED BY
NIHR | National Institute for Health Research

The University Of Sheffield. Clinical Trials Research Unit.

FIRST

NHS University Hospitals of Derby and Burton NHS Foundation Trust

Prospective randomised controlled trial comparing three splints for finger flexor tendon repairs **(FIRST)** Interview Sub-study

Qualitative Interview Audio Consent Form

Participant Identification Number: S _ _ / _ _ _

Name of Researcher:

Note: Prior to the consent process, the researcher will have confirmed that the participant has a copy of the study information sheet to hand (if not, this can be read to them) and answered any questions raised. *Instructions for the researcher are underlined*.

Before we start the interview, I need to confirm with you that you understand what is involved and whether or not you agree to take part. I will be recording this process. Is that alright with you? *(If **yes**, start recording and proceed as follows)*

I am going to read some statements to you. After each one, please answer 'yes' if you agree with the statement; or, 'no' if you do not agree with the statement.

**By completing each box and signing this form:**		***Enter Response*** Y/N
1.	I confirm that I have read the interview information sheet dated.................... (version............) for the above study. I have had the opportunity to consider the information, ask questions and have had these answered satisfactorily. *(Wait for response)*	
2.	I understand that my participation is voluntary and that I am free to withdraw at any time without giving any reason, without my medical care or legal rights being affected. *(Wait for response)*	
3.	I understand that the interview will be audio-recorded, transcribed, and used for research purposes. *(Wait for response)*	
4	I understand that the audio-recording will be stored at the University of Sheffield and destroyed at the end of the study. *(Wait for response)*	
5	I understand that quotations from the interview may be used in study in reports but these will not be identifiable. *(Wait for response)*	
6.	I agree for information collected during the interview to be used to support other research in the future, and may be shared anonymously with other researchers. *(Wait for response)*	

Please turn over

7. I agree to take part in the interview. *(Wait for response)*

8. I would like to receive a summary of the findings at the end of the study.

   Please send the summary by: email OR post (please circle preferred)
   (Wait for response)

*(If responses 1-5 are '**yes**', proceed as follows)*
Thank you. Now I need you to state your name for me so that it is recorded with this consent information *(wait for response and print name)*

The date today is:

Please can you confirm that this is the date on which you have given your permission *(wait for response and complete the section below)*

Name of person taking audio consent

Date d d m m y y y y

Signature
